# Local Epigenomic Data are more Informative than Local Genome Sequence Data in Predicting Enhancer-Promoter Interactions Using Neural Networks

**DOI:** 10.3390/genes11010041

**Published:** 2019-12-29

**Authors:** Mengli Xiao, Zhong Zhuang, Wei Pan

**Affiliations:** 1 Division of Biostatistics, University of Minnesota, Minneapolis, MN 55455, USA; xiaox345@umn.edu; 2 Department of Electrical and Computer Engineering, University of Minnesota, Minneapolis, MN 55455, USA; zhuan143@umn.edu

**Keywords:** boosting, convolutional neural networks, deep learning, feed-forward neural networks, machine learning

## Abstract

Enhancer-promoter interactions (EPIs) are crucial for transcriptional regulation. Mapping such interactions proves useful for understanding disease regulations and discovering risk genes in genome-wide association studies. Some previous studies showed that machine learning methods, as computational alternatives to costly experimental approaches, performed well in predicting EPIs from local sequence and/or local epigenomic data. In particular, deep learning methods were demonstrated to outperform traditional machine learning methods, and using DNA sequence data alone could perform either better than or almost as well as only utilizing epigenomic data. However, most, if not all, of these previous studies were based on randomly splitting enhancer-promoter pairs as training, tuning, and test data, which has recently been pointed out to be problematic; due to multiple and duplicating/overlapping enhancers (and promoters) in enhancer-promoter pairs in EPI data, such random splitting does not lead to independent training, tuning, and test data, thus resulting in model over-fitting and over-estimating predictive performance. Here, after correcting this design issue, we extensively studied the performance of various deep learning models with local sequence and epigenomic data around enhancer-promoter pairs. Our results confirmed much lower performance using either sequence or epigenomic data alone, or both, than reported previously. We also demonstrated that local epigenomic features were more informative than local sequence data. Our results were based on an extensive exploration of many convolutional neural network (CNN) and feed-forward neural network (FNN) structures, and of gradient boosting as a representative of traditional machine learning.

## 1. Introduction

Non-coding genome sequences, including enhancers, promoters, and other regulatory elements, play important roles in transcriptional regulation. In particular, through enhancer-promoter interactions (i.e., physical contacts), the enhancers and promoters coordinately regulate gene expression. Although enhancers can be distal from promoters in the genome, they are brought close to, and possibly in contact with, each other in the 3-D space through chromatin looping. Some enhancers even bypass adjacent promoters to interact with the target promoters in response to histone or transcriptional modifications on the genome. An accurate mapping of such distant interactions is of particular interest for understanding gene expression pathways and identifying target genes of GWAS loci [1,2,3].

Experimental methods based on chromosome conformation capture (3C, 4C, and Hi-C) or extensions that incorporate ChIP-sequencing such as paired-end tag sequencing (ChIA-PET) are, however, costly, and the results are only available for a few cell types [4,5,6,7]. Computational tools offer an alternative by utilizing various DNA sequence and/or epigenomic annotation data to predict EPIs with machine learning models built from experimentally obtained EPI data [8,9,10,11].

Whalen, et al. [11] reported that a gradient boosting method, called TargetFinder, accurately distinguished between interacting and non-interacting enhancer-promoter pairs based on epigenomic profiles. They included histone modifications and transcription factor binding (based on ChIP-seq), and DNase I hypersensitive sites (DNase-seq) with a focus on distal interaction (>10 kb) in high resolution. The idea was further extended to predict EPIs solely from local DNA sequence data and achieved high prediction accuracy [12,13,14].In particular, convolutional neural networks (CNNs), known for capturing stationary patterns in data with successful applications in image and text recognition [15,16], were shown to perform well in predicting EPIs based on DNA sequence alone. A natural question is whether CNNs can further improve the prediction performance with regional epigenomic features. It is also noted that for DNA sequence data, differing from for images, a simple CNN model seemed to perform well [14]; a similar conclusion was drawn for other biological data [17].

On the other hand, two recent studies pointed out an experimental design issue of randomly splitting the original data for training and testing as adopted by most, if not all, previous studies: many promoters interact with multiple, possibly overlapping, enhancers concurrently. Such pairs, some in the training data while others in the test data, are not independent, leading to possibly over-fitting a model and over-estimating its predictive performance [18,19] Since promoters primarily interact with enhancers on the same chromosome, the problem could be avoided by having different chromosomes split into the training and test data. Based on such a valid training and test data, Xi and Beer [19] concluded that local epigenomic features around enhancers and promoters alone were not informative enough to predict EPIs and they suggested to re-evaluate similar studies on EPI prediction. Although combining local epigenomic features and sequence data was found to improve prediction in a recent study [20] it was based on a random splitting of the whole dataset into training and test data, thus possibly suffering from inflated performance. After correcting for such experimental design bias, we would like to address two important and interesting questions: (1) whether or not local enhancer-promoter sequences are more informative than corresponding local epigenomic features; (2) whether or not we can gain more information by combining local sequence and epigenomic annotations.

Here, we report our extensive study of local sequence and epigenomic data for predicting long-range EPIs; in addition to more recent and popular CNNs and gradient boosting as adopted in most of prior studies, we also considered more traditional feed-forward neural networks (FNNs) [21,22]. After avoiding the previous experimental design issue, we found that local sequence data alone were insufficient to predict EPIs well; in comparison, local epigenomic signals, albeit not highly predictive either, were more informative than sequence data. Furthermore, combining local sequence data with local epigenomic profiles did not improve over using local epigenomic data alone. These results may be useful for future studies.

## 2. Materials and Methods

In this paper, we primarily studied one of the six cell lines, K562, with a large sample size and top performance in previous studies among the six cell lines, to investigate within-cell-line predictive performance [11]. The sample size was 41,477 in total with 1977 positive cases (i.e., interactions) and 39,500 negative cases.

### 2.1. Data

**Figure 1:**
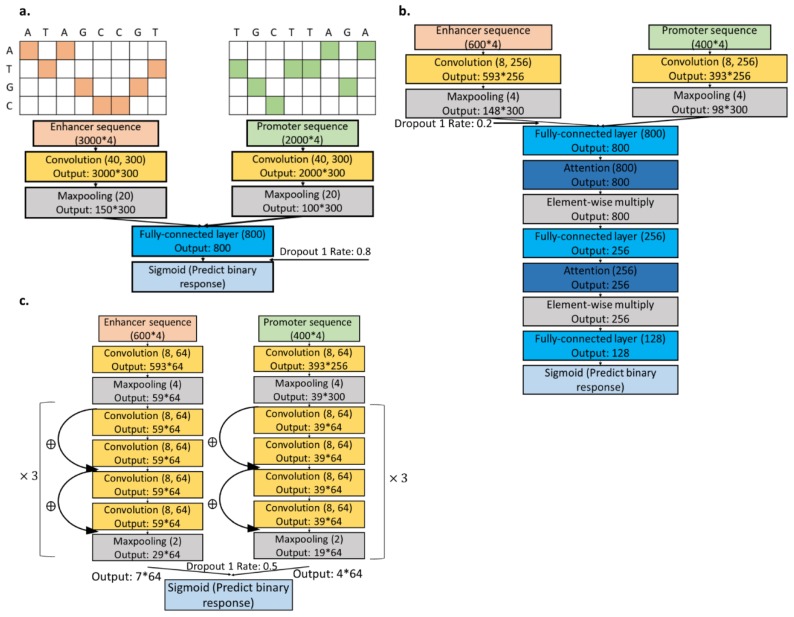
Sequence model structures. (**a**) The structure followes a previously reported simple convolutional model [14]. We used shorter input sequences, each centered as the SPEID data, with attention modules (**b**) or ResNet (**c**).

**Figure 2:**
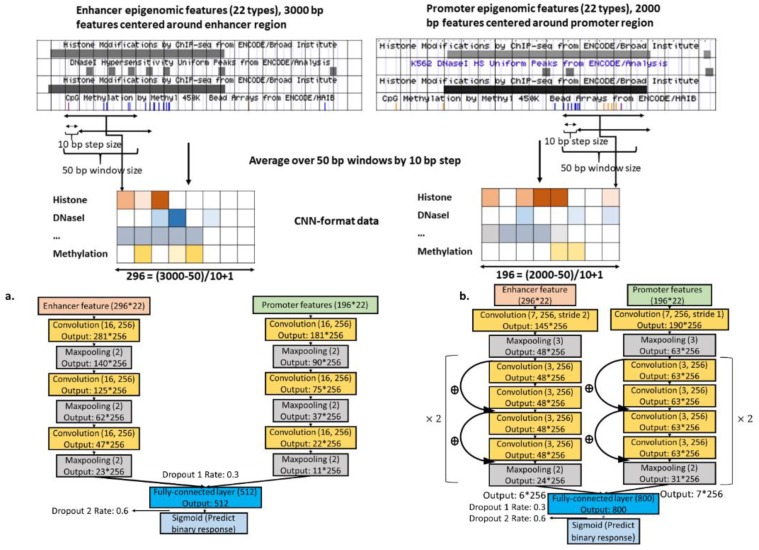
Epigenomics data preparation and CNN model structures. Different genomic annotation data type was denoted with different color. (**a**) Basic CNN structure; (**b**) ResNet CNN structure.

**Figure 3:**
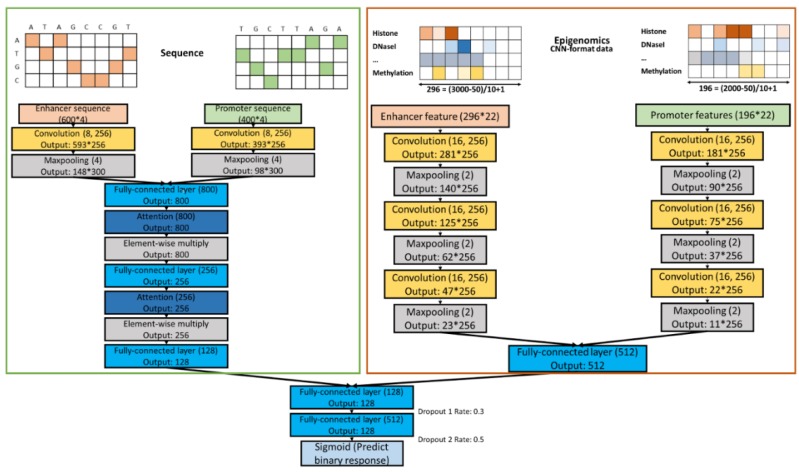
Combined model structure with the sequence and epigenomic models.

**Figure 4:**
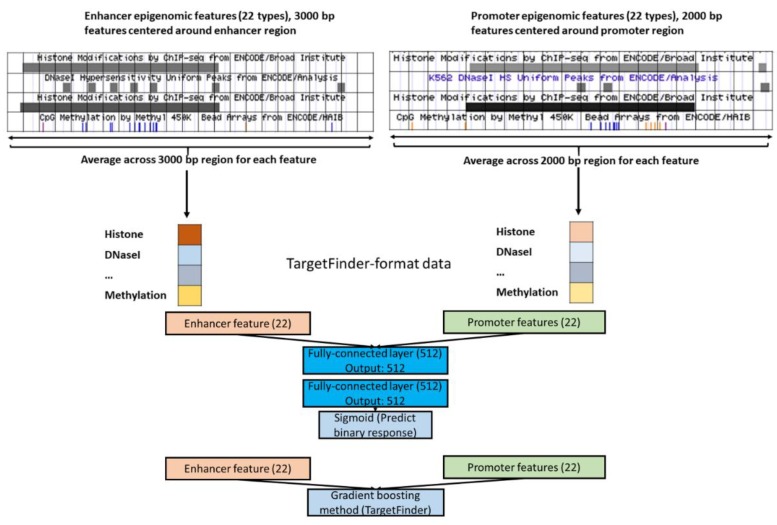
TargetFinder-format (epigenomic) data and the feed-forward neural network (FNN) model structure.

We retrieved the DNA sequence data packaged in the SPEID model of Singh, et al. [13] at the website http://genome.compbio.cs.cmu.edu/~sss1/SPEID/all_sequence_data.h5. Each sample represented an interacting/non-interacting pair of one-hot encoded DNA sequence centered at one enhancer (3000 bp) and one promoter (2000 bp), which were only local features compared to >10 kb distance between an enhancer and a promoter in the dataset [11] As for the local sequence data used previously [13,14], epigenomic data length was set to be 3000 bp for enhancers and 2000 bp for promoters in all epigenomic data types. We used the epigenomic features that are shared across the cell lines with most genomic features (K562, GM12878, HeLa-S3, and IMR90) based on Supplemental Table 2 in Whalen, et al. (2016). There are 22 epigenomic data types in total, including 11 histone mark peaks (H3K27ac,H3K27me3,H3K4me1, H2AZ, H3K4me2, H3K9ac, H3K4me3, H4K20me1, H3K79me2, H3K36me3, H3K9me3), 9 transcriptional factor bindings (POLR2A, CTCF, EP300, MAFK, MAZ, MXI1, RAD21, RCOR1, RFX5), DNase and methylation (ENCODE Project Consortium, 2007; Bernstein, 2010). Hence, the data dimensions were (# of samples)×3000×22 for enhancers and (# of samples×2000×22) for promoters (Figure 1, Figure 2 and Figure 3). We extracted the epigenomic data as following. According to the enhancer/promoter genome coordinates available in the TargetFinder E/P dataset, the 3000 and 2000 genome window coordinates centered around the enhancer and promoter in each pair were calculated, then the data at each base pair were retrieved via those calculated window coordinates from 22 epigenomic data files across the whole genome for cell line K562 in the BigWig format, available at the ENCODE or NIH Roadmap Epigenomic projects [23,24]. We used package pyBigWig (http://dx.doi.org/10.5281/zenodo.45238) to read in BigWig files. However, one type of epigenomic data file in the BigWig format was often measured with multiple sample replicates, but the ENCODE or Roadmap project summarized those measurements only in BED format. The two file formats contain epigenomic feature information in different genome scales. Each unit in the BED file represents a small sampled genome sub-region with experimentally measured signals, thus the same base pair may be measured multiple times in multiple and different sub-region samples, which can be combined to map a unique signal value to each base pair by available tools for the whole genome. BigWig file, however, has a 1-1 correspondence between one base pair and a signal value across the whole genome. Thus, we obtained such 1-1 map between signals and genome in BigWig format data from BED files in Whalen, et al. [11] at https://github.com/shwhalen/targetfinder/blob/master/paper/targetfinder/K562/, which came from the cleaned peak files through the ENCODE or Roadmap. The Bedtools and bedGraphToBigWig software (http://hgdownload.soe.ucsc.edu/admin/exe/linux.x86_64/) was used to merge and convert BED files to the BigWig format [25]. To compare with TargetFinder, where data was summarized by computing the mean signal value across the whole local region of interest (3000/2000 bp), we also took the mean of the local epigenomic signals across each 3000/2000-bp window Later we will refer it as the TargetFinder-format data, which was 2-dimensional (Figure 4), and not suitable for CNNs. The epigenomic data were large to read in (13–23 GB) and often sparse in a 3000/2000-bp interval. Even when not sparse, the signal values often remained the same across multiple base pairs. The redundant and noisy data unnecessarily increased the number of parameters in prediction models. Therefore, we considered averaging the data signals through different sliding-window sizes and step sizes. Through the validation performance of CNNs, we found that a window/bin size of 50 and step size of 10 performed well among non-summarized and other forms of summarized data (Table S1). The data were later input into CNNs, hence we call it CNN-format data (Figure 2). After the sliding-window operation, the CNN-format data were reduced to 1–2 GB.

To combine sequence and epigenomic data sources, a one-to-one mapping from enhancer-promoter pairs in the TargetFinder dataset to SPEID sequences was established. Although the exact procedure in the Singh, et al. [13] about sequence data generation from the TargetFinder E/P dataset was not available online, we inferred the relationship by matching sequences. Given the provided enhancer/promoter locations on the genome in TargetFinder, we first retrieved the enhancer and promoter sequence segments from the hg19 reference genome at http://genome.ucsc.edu/cgi-bin/das/hg19/dna?segment=chr1:6454864,6455189 (e.g., an enhancer was located on chromosome 1 from position 6454864 to 6455189 bp), then searched a matching SPEID enhancer/promoter sequence.

We always used the data on chromosomes 8 and 9 as the validation data; we used each of the remaining chromosome in turn as a test dataset while the other chromosomes (not the three chromosomes used as the validation and test data) as the training data. This avoided the bias issue of the original random data splitting [19].

### 2.2. Designing Neural Networks for Utilizing Enhancer and Promoter Features

Our main goal was to construct predictive CNN models for detecting distal EPIs from sequence and epigenomic data, separately or combined. CNNs for sequence and epigenomic annotations were built separately, and we constructed another model to combine features from the two sources. In either sequence or epigenomics CNN, we considered two separate branches for enhancers and promoters respectively, then concatenated the features later. This followed from that enhancers and promoters are expected to have different sequence motifs or epigenomic profiles, as shown in previously designed CNNs [13,14,20].

### 2.3. Sequence CNNs.

Figure 1 shows different CNN models for sequence data after splitting the data by chromosomes to prevent non-independent training, validation, and test data. We conducted an extensive evaluation of CNNs with varying structures and parameters (Table 1 and Table S2). We first tested the performance of the CNN in Zhuang, et al. [14], and then added an attention module to utilize the sequence structure and focus only on the middle part of the input sequence [26]. Moreover, we tested on the use of the Residual Neural Network (ResNet) architecture for the data (Table 2) [27,28].

### 2.4. Epigenomics CNNs

A 4-layer CNN (called basic CNN) and a 10-layer ResNet are shown in Figure 2, chosen by the validation performance to predict EPIs based on epigenomic data (Table 3 and Table S2). We also implemented a few other newer CNN models, including Inception and Capsule Networks because of their improved performance in image applications [29,30]. Likely due to limited epigenomic data, their results were less competitive than simpler CNNs; they provided no higher prediction power than random guessing (result not shown).

### 2.5. Combined Models 

To combine the features from two data sources, we constructed 1 or 2 hidden layers (Figure 3 and Table S2). We saved high-level features extracted from the previous CNNs for sequence and epigenomic data, then combined them together as input to the first hidden layer in the combined model. We also tested more complex combination schemes but found no performance improvement; for example, we trained and combined the individual models simultaneously, instead of doing the two steps sequentially.

### 2.6. Designing FNNs.

Since an FNN can take epigenomic data as input in the same format as that of TargetFinder (i.e., TargetFinder-format data), we were interested to know if such a simple FNN could outperform TargetFinder or other CNNs. After tuning the model parameters through the validation dataset, we chose a 2-layer FNN shown in Figure 4.

An FNN can take both TargetFinder-format data and CNN-format data as input, and our analysis showed an FNN with the same structure performed better with the TargetFinder-format data than that with the CNN-format data (Table S3). Therefore, we further explored the performance of basic CNNs shown in Figure 2 and FNNs shown in Figure 4 in comparison to gradient boosting as implemented in TargetFinder [11]. We not only implemented neural networks for K562 cell line, but also expanded our evaluations to other cell lines (GM12878, HeLa-S3, IMR90). The training configuration was similar to that for CNNs.

### 2.7. CNN Training in an Imbalanced-Class Scenario

For each CNN model in our study, the number of model parameters was much larger than the available training sample size, which might cause overfitting and numerical instability. In addition, there was a high ratio of class imbalance (positive:negative = 1:20), posing another challenge to training predictive models. Different training techniques were adopted to address these problems. Batch normalization was added after each layer to stabilize gradient updates and reduce the dependence on initialization [31]. The neural network weights were initialized from the glorot uniform distribution: U(-sqrt(6/(# of input weights+# of output weights)), sqrt(6/(# of input weights+# of output weights))) or He’s normal distribution in ResNet for all layers [32,33]. The parameters were estimated through the Adam optimizer [34]. The learning rate and batch size were tuned across multiple grid values (Table S2). We used the weight decay and dropout for regularization [35], and more details about our final training parameters for all main CNNs are in Table 1. Another regularization technique is the early stopping that was determined by the validation data on chromosomes 8 and 9; the training was stopped if no improvement of the validation F1 score was observed over 10 epochs. Final evaluation on the test data used the model with the highest validation F1 score across all the training epochs before early stopping.

In order to address the problem of highly imbalanced classes in the training data, we used a weighted objective function—a binary cross entropy for this imbalanced dataset. In the training data, the weight for positive or negative pairs was given by the ratio of a half of the sample size over the number of positive or negative pairs. We also attempted to achieve a balanced training sample by augmenting the training data through oversampling positive pairs (i.e., the minority class) or down-sampling negative pairs in generating batched training data for neural networks, but the test performance was not significantly better than using a weighted objective function (with a *p* value of 0.4097 from the paired *t*-test; Table S4).

### 2.8. Evaluating Model Performance

With local sequence and 22 types of epigenomic features in our highly imbalanced dataset, the prediction performance was assessed through an Area Under Receiver Operating Characteristic (AUROC) with test data. Note that we had 21 test datasets corresponding to 21 chromosomes besides chromosomes 8 and 9, which were used as the validation data. Since the numbers of enhancer-promoter pairs varied with chromosomes, we reported a weighted average and standard deviation of the AUROC’s across 21 chromosomes, where the weight was the (# of samples on each chromosome)/(total # of samples on 21 chromosomes).

To examine if either sequence or epigenomics model prediction performance could benefit from the other data source, we also evaluated the chromosome-wise test AUROCs for the combined model structure in Figure 4. Data splitting and training configurations (including tuning both structural and training parameters) followed exactly as before. We combined the models with the highest mean AUROCs from the two data sources respectively in Table 4, which were the sequence attention model (central region) and epigenomics basic CNN model. We also conducted the paired *t*-test across the 21 test chromosomes for the difference between two methods (with a null hypothesis $H_{0}$ that two methods gave the same test AUROC for each chromosome). The *p* values reported here were not adjusted for multiple comparisons, but would remain significant (or insignificant) after the Bonferroni adjustment.

### 2.9. Implementation and Code Availability

All the neural networks below were implemented in Keras (2.0.9) with Tensorflow (1.4.0) on a GPU server (NVIDIA Telsa K40 GPU). The choice of the parameters for gradient boosting followed TargetFinder (https://github.com/shwhalen/targetfinder), and we chose the # of trees through the validation dataset. Gradient boosting was implemented with Python Sklearn. The code is available at https://github.com/menglix/EPI.

## 3. Results

### 3.1. Local Epigenomic Features Were more Informative than Local Sequence Data in Predicting EPIs

**Figure 5:**
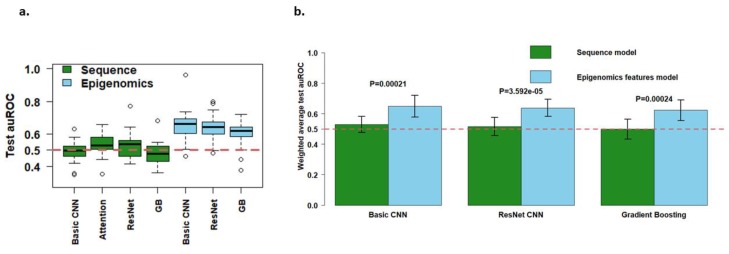
Comparison on the weighted average Area Under Receiver Operating Characteristic (AUROC) between the sequence and epigenomics models. (**a**) Boxplot of test AUROCs for all sequence and epigenomic models across 21 chromosomes; (**b**) Mean test AUROC comparison between the sequence and epigenomics models. The bars are ±1 weighted standard deviation around the weighted mean AUROC. *p* values are from the paired *t*-test with $H_{0}$: The test AUROCs are the same for the sequence and epigenomics models. *p* values are not adjusted for multiple comparison but remained significant after the Bonferroni adjustment.

Although previous studies found using sequence data yielded as good or even better prediction performance as/than using epigenomic data [13,14], this trend was reversed after a valid data splitting scheme was applied (Methods). Figure 5a shows that using (local) epigenomic data outperformed using (local) sequence data across all test chromosomes for each of multiple prediction models. We also compared the performance of the two data sources with similar models side by side in Figure 5b, where the basic CNN, ResNet CNN and gradient boosting were customized to the two data sources during the training process. *p*-values of the paired *t*-test (for each chromosome) to compare model weighted average AUROC were all <0.0002, suggesting that the local epigenomics data gave a statistically significant and stronger performance than the local sequence data.

Interestingly, while the test AUROC’s for the sequence models were very close to 0.5, corresponding to random guessing, the epigenomic models achieved a notable difference from 0.5 (Figure 5a,b). This again suggests a better performance of using the epigenomics data among all prediction models implemented here. Furthermore, the AUROC’s of the epigenomics models had similar standard deviations across chromosomes to those of the sequence models, and even minus one standard deviation of the mean AUROC of the epigenomics models was above the sequence models’ average AUROC for each of the 3 scenarios in Figure 5b. Since the epigenomics models’ AUROCs were significantly different from that of the sequence models, which were comparable to random guessing (0.5), local epigenomics features were still predictive of EPIs, though the performance was much inflated in previous studies [11,19,20]. Our finding also demonstrated that without appropriate data splitting (or generally valid experimental design), any EPI prediction results and downstream motif analyses with DNA sequence data should be interpreted with caution.

### 3.2. Combining Epigenomic and Sequence Data: Do We Gain Additional Information?

**Figure 6:**
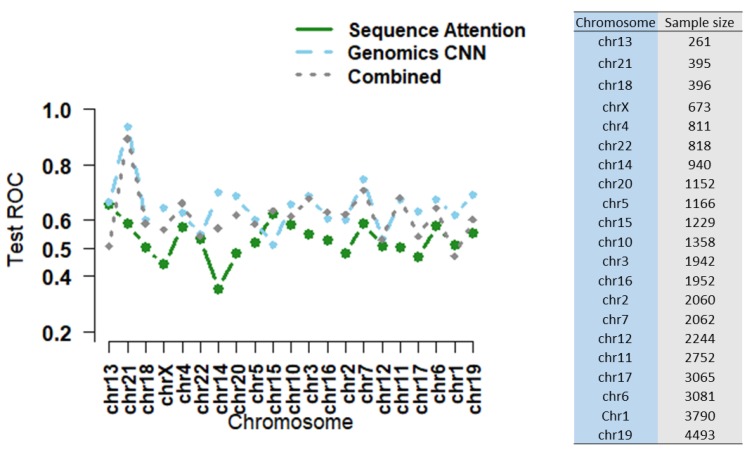
The combined CNN model performance as compared to the sequence and epigenomics CNNs across 21 test chromosomes with the sample size in ascending order.

As shown in Table 4 and Figure 6, the combined model showed an improved mean AUROC over that of the sequence model (AUROC of 0.603 vs. 0.529), though the result was expected as epigenomics features showed a higher predictive power than sequences from Figure 5. Still, our finding was consistent with other publications showing integrating epigenomic features with sequence data improved the performance over using sequence data alone in predicting epigenomics-related features with fully-connected layers or recurrent neural network [20,36,37]. However, the performance was not enhanced over that of the epigenomics model (AUROC of 0.603 vs. 0.648).

### 3.3. Are more Complex Structures and more Parameters Needed for High-Dimensional Data Input?

**Figure 7:**
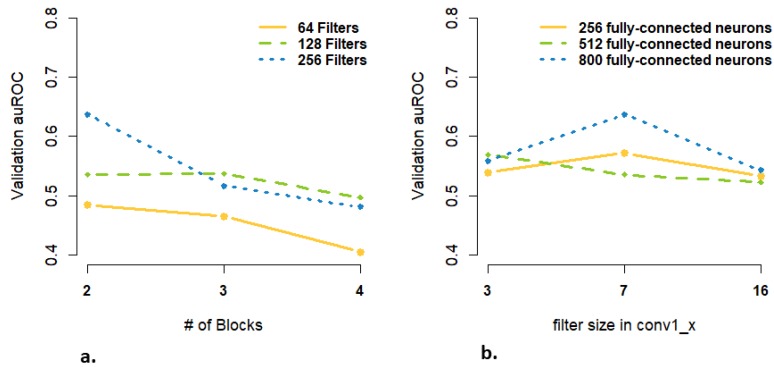
Validation AUROCs for epigenomics ResNet CNNs with various configurations in Table 3: (**a**) the number of ResNet blocks and number of filters for each convolution; (**b**) the filter/kernel size in the first layer and number of fully-connected neurons in the last layer.

Our input data for CNN models were relatively high-dimensional (with the enhancer sequence and epigenomic features of dimensions of 3000 × 4 and 296 × 22 respectively). Through our extensive explorations of various CNN architectures/structures, we observed that both epigenomic data- and sequence data-based prediction models performed better with simpler CNNs (Table 2 and Table 3). At the same time, we still needed a large number of parameters in each layer, leading to over-parametrized models. For example, in the epigenomic ResNet model, although the highest validation AUROC required only 2 blocks (i.e., 8 convolution layers in Table 3), it requires a large number of filters (256) according to Figure 7. Several lines of evidence during our model tuning supported our conclusions. First, our observed optimized numbers of layers in CNNs were small, in contrast to deep learning models in image recognition and other applications (Table 2 and Table 3). This was perhaps partly due to some inherent differences between the biological data used here and images, the latter of which can be represented by a hierarchy of from low- to high-level features requiring a large number of layers or deep neural networks [38]. Given the complexity behind regulatory mechanism of enhancer and promoter, a large number of parameters are still needed for capturing regional/local dependencies and interactions in sequence and epigenomic data (Table 2 and Table 3 and Figure 7). Second, among the models for the same data source, probably due to the small number of layers, we noted that adding skip connections did not show a clear advantage over a basic CNN in predicting EPIs (Figure 5a,b). Skip connections as adopted in ResNets were reported to improve prediction performance, partly by better optimizing a deep CNN during the training process [28]. In our work, we observed the optimal number of layers (in a ResNet) at 8 (Figure 1 and Table 3), which was much less than that of a typical ResNet (with 18, 34 or even 1000 layers).

Finally, besides modeling enhancer or promoter regulatory machineries separately, a large number of parameters was also desirable for characterizing complex interaction patterns between an enhancer and a promoter. We showed that a ResNet without any fully connected layer after the concatenation of the enhancer and promoter branches performed worse than models with fully-connected layers (Table S4), although the result is not significant (Paired *t*-test *p* value: 0.2546). In addition, as Figure 7b. demonstrates, 800 fully-connected neurons in the ResNet CNN, corresponding to a higher number of parameters, had the highest validation AUROC. To further illustrate the necessity of having enhancer and promoter as separate branches for CNN models, we also tried inputting aggregated enhancer and promoter epigenomics data at the beginning of the basic CNN model, where the interactions are modeled at the beginning through neural networks (Table 3). As the number of parameters is larger than modeling enhancer and promoter as separate branches, the weighted average AUROC was slightly better but not significant (Table S4; Paired *t*-test *p* value: 0.4255), which suggested a more over-parametrized model did not deteriorate the model performance.

### 3.4. Epigenomics Feed-Forward Neural Networks (FNNs) Performed Better than Gradient Boosting

**Figure 8:**
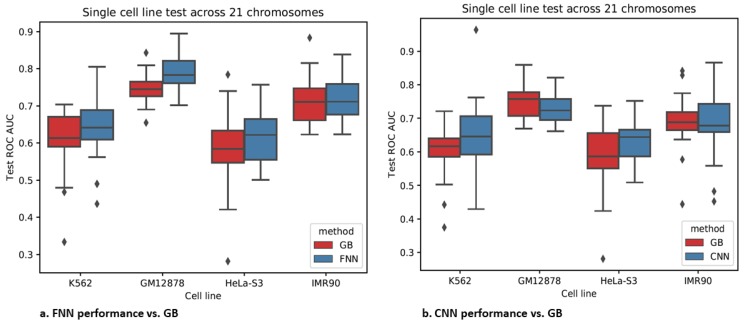
FNN and CNN prediction performance for the same data format as that for GB.

Both FNNs and CNNs had a higher or comparable test AUROC than gradient boosting with either of the data formats (TargetFinder-format and CNN-format) across the 21 test chromosomes for most cell lines (Figure 8 and Table S5). In addition, the training time of FNNs by leveraging GPUs was faster than gradient boosting (e.g., 1–2 min vs. 3–10 min in GB). Little evidences from Table S5 supported that either neural network or gradient boosting was capable of cross-cell-line prediction.

The FNNs performed better than the CNNs in cell lines GM12878 and IMR90 and similar to the CNNs in other two cell lines with slightly lower standard deviations than the CNNs. Although valuable spatial dependency information in a region might be retained in the CNN-format data, the increased data dimension and high noise levels might discount the corresponding benefits. As a side note, the FNNs were still over-parametrized with the input data of dimension only 44 and the training sample size of less than 40,000.

## 4. Conclusions

Through an extensive evaluation of the use of various neural networks, especially convolutional neural networks (CNNs), on predicting enhancer-promoter interactions (EPIs), we demonstrated that local epigenomic features were more predictive than local sequence data. In contrast to most previous studies on EPI prediction, we reached our conclusions by holding out data from one or more whole chromosomes as training, validation, and test data respectively, avoiding biases associated with random partitioning of enhancer-promoter pairs as training, validation, and test data [19]. We also did not find much predictive gain in integrating local features from the two data sources, perhaps because local sequences were not informative enough for a higher prediction accuracy. We emphasize that, although our findings suggest that local DNA sequence data may not be sufficient to well predict EPIs, a new study has shown some promising results of using mega-base scale sequence data incorporating large-scale genomic context [39]; this is in agreement with improved prediction performance of including not only local epigenomic features of an enhancer and a promoter, but also the window region between them [40]. More studies are warranted.

## Figures and Tables

**Table 1:** Parameters for convolutional neural network (CNN) models.

	Batch Size	Learning Rate	L2 Weight Decay in the Convolution	Dropout 1	Dropout 2
Sequence					
Zhuang et al., 2019 [14] (Basic CNN)	64	1.0 × 10^−5^	1.0 × 10^−6^	0.2	0.8
Attention CNN	64	1.0 × 10^−5^	2.0 × 10^−5^	0.2	None
ResNet CNN	200	1.0 × 10^−5^	2.0 × 10^−5^	0.5	None
Epigenomics					
Basic CNN	200	1.0 × 10^−6^	1.0 × 10^−5^	0.3	0.6
ResNet CNN	64	1.0 × 10^−3^	1.0 × 10^−5^	0.3	0.6
FNN	200	1.0 × 10^−6^	1.0 × 10^−5^	0.7	None
Combined model	200	1.0 × 10^−5^	1.0 × 10^−6^	0.3	0.5

**Table 2:** Sequence model structures.

Block Name	Simple/Basic CNN	Attention	ResNet without Fully-Connected Layer
Input	$\left[\begin{array}{c} \mathrm{Enhancer}:3000\times 4\\ \mathrm{Promoter}:2000\times 4 \end{array}\right]$	$\left[\begin{array}{c} \begin{array}{c} \mathrm{Enhancer}:600\times 4\\ \mathrm{Promoter}:400\times 4 \end{array} \end{array}\right]$	$\left[\begin{array}{c} \begin{array}{c} \mathrm{Enhancer}:600\times 4\\ \mathrm{Promoter}:400\times 4 \end{array} \end{array}\right]$
conv1_x	$\left[\begin{array}{c} 40\times 1,300\\ \max\mathrm{pooling}\left(20\times 1\right) \end{array}\right]$	$\left[\begin{array}{c} 8\times 1,256\\ \max\mathrm{pooling}\left(4\times 1\right) \end{array}\right]$	$\left[\begin{array}{c} 8\times 1,64\\ \max\mathrm{pooling}\left(10\times 1\right) \end{array}\right]$
conv2_x			$\left[\begin{array}{c} 8\times 1,64\\ 8\times 1,64 \end{array}\right]\times 2$
conv3_x			$\left[\begin{array}{c} 8\times 1,64\\ 8\times 1,64 \end{array}\right]\times 2$
conv4_x			$\left[\begin{array}{c} 8\times 1,64\\ 8\times 1,64 \end{array}\right]\times 2$
Concatenate branches	800-d fc, sigmoid	$\left[\begin{array}{c} \begin{array}{c} 800-d\mathrm{fc}\\ 800-d\mathrm{attention}\\ \mathrm{multiply}\mathrm{the}\mathrm{outputs} \end{array}\\ 256-d\mathrm{fc}\\ 256-d\mathrm{attention}\\ \mathrm{multiply}\mathrm{the}\mathrm{outputs}\\ 128-d\mathrm{fc}\\ \mathrm{Sigmoid} \end{array}\right]$	$\left[\begin{array}{c} \mathrm{no}\mathrm{avg}\mathrm{pooling}\left(\mathrm{output}\mathrm{dimesnion}\mathrm{is}11\right)\\ \mathrm{Sigmoid} \end{array}\right]$
# of parameters	60,100,402	51,345,538	605,452

**Table 3:** Epigenomic model summary.

Block Name	Basic CNN (2 Branches)	Basic CNN (1 Branch)	ResNet with Fully-Connected (fc) Layer	ResNet without Fully-Connected (fc) Layer
Input	$\left[\begin{array}{c} \mathrm{Enhancer}:296\times 22\\ \mathrm{Promoter}:196\times 22 \end{array}\right]$	$\left[\begin{array}{cc} \begin{array}{c} \mathrm{Enhancer}\\ +\\ \mathrm{Promoter} \end{array}: & 492\times 22 \end{array}\right]$	$\left[\begin{array}{c} \mathrm{Enhancer}:296\times 22\\ \mathrm{Promoter}:196\times 22 \end{array}\right]$	$\left[\begin{array}{c} \mathrm{Enhancer}:296\times 22\\ \mathrm{Promoter}:196\times 22 \end{array}\right]$
conv1_x	$\left[\begin{array}{c} 16\times 1,256\\ \max\mathrm{pooling}\left(2\times 1\right) \end{array}\right]\times 3$	$\left[\begin{array}{c} 16\times 1,256\\ \max\mathrm{pooling}\left(2\times 1\right) \end{array}\right]\times 3$	$\left[\begin{array}{c} 7\times 1,64,\mathrm{stride}2\mathrm{or}1*\\ \max\mathrm{pooling}\left(3\times 1\right) \end{array}\right]$	$\left[\begin{array}{c} 7\times 1,64,\mathrm{stride}2\mathrm{or}1*\\ \max\mathrm{pooling}\left(3\times 1\right) \end{array}\right]$
conv2_x			$\left[\begin{array}{c} 3\times 1,256\\ 3\times 1,256 \end{array}\right]\times 2$	$\left[\begin{array}{c} 3\times 1,128\\ 3\times 1,128 \end{array}\right]\times 2$
conv3_x			$\left[\begin{array}{c} 3\times 1,256\\ 3\times 1,256 \end{array}\right]\times 2$	$\left[\begin{array}{c} 3\times 1,128\\ 3\times 1,128 \end{array}\right]\times 2$
conv4_x				$\left[\begin{array}{c} 3\times 1,128\\ 3\times 1,128 \end{array}\right]\times 2$
conv5_x				$\left[\begin{array}{c} 3\times 1,128\\ 3\times 1,128 \end{array}\right]\times 2$
Concatenate branches	$\left[\begin{array}{c} 512-d\mathrm{fc}\\ \mathrm{Sigmoid} \end{array}\right]$	$\left[\begin{array}{c} \mathrm{No}\mathrm{concatenation}\\ 512-d\mathrm{fc}\\ \mathrm{Sigmoid} \end{array}\right]$	$\left[\begin{array}{c} \begin{array}{c} \mathrm{avg}\mathrm{pooling}\left(2\times 1\right)\\ 800-d\mathrm{fc} \end{array}\\ \mathrm{Sigmoid} \end{array}\right]$	$\left[\begin{array}{c} \mathrm{avg}\mathrm{pooling}\\ \mathrm{Sigmoid} \end{array}\right]$
# of parameters	8,838,145	12,244,481	5,915,841	1,625,985

* stride 1 for promoter and stride 2 for enhancer.

**Table 4:** Epigenomics and sequence model performance.

Epigenomics Model	Mean AUROC	Standard Deviation of AUROC	Sequence Model	Mean AUROC	Standard Deviation of AUROC
Simple convolution/Basic sequence CNN (Zhuang et al., 2019 [14]; original region)	0.500	0.0500	Basic CNN	0.648	0.0704
Attention CNN (central region)	0.529	0.0533	ResNet CNN	0.638	0.0568
ResNet CNN (central region)	0.515	0.0596	Gradient Boosting	0.622	0.0676
Gradient Boosting (central region)	0.494	0.0557	Combined model	0.603	0.0703
Gradient Boosting (original region)	0.499	0.0654			

## Supplementary Materials

The following are available online at https://www.mdpi.com/2073-4425/11/1/41/s1, Table S1: Performance of CNNs with varying window and step sizes for the TargetFinder dataset without correct training/validation/test data splitting for cell line GM12878. Table S2: Parameter search grids for CNN models. Table S3: FNN performance comparison between two data formats using chromosome 1 as the test data (with the results for the training data in parentheses). Table S4: Performance summary of additional epigenomics CNN models. Table S5: The single-cell-line and cross-cell-line mean (SD) test AUROCs across each of the 21 test chromosomes for Gradient Boosting (GB) in comparison with the CNNs and FNNs with the same data format.
