# Supplementary material for: Local Epigenomic Data are more Informative than Local Genome Sequence Data in Predicting Enhancer-Promoter Interactions Using Neural Networks
Source: Genes (Basel). 2019 Dec 29;11(1):41. doi: 10.3390/genes11010041 (PMC7016741; doi:10.3390/genes11010041)
Supplement: Supplementary file 1 [file genes-11-00041-s001.pdf]

Supplementary Materials:

Table S1: Performance of CNNs with varying window and step sizes for the TargetFinder dataset without correct training/validation/test data splitting for cell line GM12878. We used the GM12878 cell line, instead of cell line K562, to prevent possible overfitting for the latter. The mean and standard deviation (S.D.) of test AUROC were summarized over 10 different initial values in CNNs, so that the S.D.s of test AUROC are small enough for an accurate comparison among the mean test AUROCs for each Window × Step combination.

Window × Step (bp)	Input enhancer size	Input promoter size	Mean test AUROC	S.D. of test AUROC
25×5	596	396	0.927	0.007
25×10	298	198	0.934	0.006
25×20	149	99	0.919	0.010
50×5	591	391	0.927	0.011
50×10	296	196	0.931	0.006
50×20	148	98	0.920	0.009
50×50	60	40	0.914	0.010
100×5	581	381	0.922	0.008
100×10	291	191	0.932	0.005
100×20	146	96	0.918	0.007
100×50	59	39	0.916	0.009
100×100	30	20	0.865	0.021
150×5	571	371	0.923	0.009
150×10	286	186	0.919	0.008
150×20	143	93	0.914	0.010
150×50	58	38	0.913	0.012
150×100	29	19	0.841	0.028
200×5	561	361	0.919	0.007
200×10	281	181	0.927	0.006
200×20	141	91	0.897	0.050
200×50	57	37	0.911	0.006
200×100	29	19	0.893	0.024

Table S2: Parameter search grids for CNN models.

Structural parameters					
Epigenomics					
Basic CNN	# of filters	filter size	# of fully-connected neurons		
	[128, 256]	[8, 16]	[512,800]		
ResNet CNN	# of filters	filter size in conv1_x	max pooling in conv_1	# of fully-connected neurons in concatenation	# of conv blocks
	[64,128,256]	[3,7,16]	[2,3]	[256,512,800]	[3,4,5]
Sequence*					
ResNet CNN	# of filters	filter size in residual blocks (after conv1_x)	# of conv blocks		
	[64,100,128]	[3,4,8,10]	[2,3,4,5]		
Combined model	# of kernels	# of hidden layers			
	[64,128,256,512,1024]	[1,2]			
Training parameter					
Epigenomics					
Basic CNN	initial learning rate	batch size	Dropout 1	Dropout 2	L2 weight decay in convolution
	[1e-6,5e-6,1e-5,5e-5,1e-4,5e-4,1e-3]	[32,64,128,256]	[0.0,0.2,0.3]	[0.3,0.5,0.6]	
ResNet CNN	initial learning rate	batch size	Dropout 1	Dropout 2	
	[1e-6,5e-6,1e-5,5e-5,1e-4,5e-4,1e-3]	[32,64,128,256]	[0.0,0.2,0.3]	[0.3,0.5,0.6]	
Sequence*					
ResNet CNN	initial learning rate	batch size			
	[1e-6,5e-6,1e-5,5e-5,1e-4,5e-4,1e-3]	[32,64,128,256]			
Combined model					
	[1e-4,1e-5,1e-6]				

*Basic CNN model configuration follows Zhuang et al. (2019)

Table S3: FNN performance comparison between two data formats using chromosome 1 as the test data (with the results for the training data in parentheses).

	CNN-format data	TargetFinder-format data
K562		
AUROC	0.691 (0.928)	0.718 (0.874)
GM12878		
AUROC	0.782 (0.963)	0.821 (0.848)
HeLa-S3		
AUROC	0.618 (0.922)	0.648 (0.704)
IMR 90		
AUROC	0.645 (0.990)	0.677 (0.749)

Table S4: Performance summary of additional epigenomics CNN models.

Epigenomics model	Mean AUROC	Standard deviation of AUROC	# of parameters
Basic CNN	0.648	0.0704	8,838,145
Basic CNN (without batch)	0.648	0.0748	8,838,145
Basic CNN (Concatenated at the beginning)	0.649	0.0660	12,244,481
ResNet CNN	0.638	0.0568	5,915,841
ResNet CNN without fully-connected layer	0.620	0.0653	1,625,985

Table S5: The single-cell-line and cross-cell-line mean (SD) test AUROCs across each of the 21 test chromosomes for Gradient Boosting (GB) in comparison with the CNNs and FNNs with the same data format.

CNN					CNN-format GB				
Train\Test	K562	GM12878	HeLa-S3	IMR90	Train\Test	K562	GM12878	HeLa-S3	IMR90

K562	0.648(0.0704)	0.573(0.0274)	0.516(0.0161)	0.471(0.0245)	K562	0.600(0.0869)	0.475(0.0406)	0.524(0.0191)	0.492(0.0254)
GM12878	0.561(0.0142)	0.729(0.045)	0.509(0.0136)	0.465(0.0250)	GM12878	0.620(0.0142)	0.750(0.0502)	0.571(0.0122)	0.522(0.0368)
HeLa-S3	0.529(0.0191)	0.490(0.0392)	0.635(0.0625)	0.481(0.0196)	HeLa-S3	0.460(0.0165)	0.404(0.0321)	0.583(0.107)	0.555(0.0455)
IMR90	0.475(0.0159)	0.421(0.0313)	0.492(0.0149)	0.677(0.0949)	IMR90	0.487(0.0118)	0.569(0.0292)	0.574(0.0185)	0.690(0.0825)
TargetFinder-format FNN					TargetFinder-foramt GB				
Train\Test	K562	GM12878	HeLa-S3	IMR90	Train\Test	K562	GM12878	HeLa-S3	IMR90
K562	0.635(0.0864)	0.483(0.0441)	0.466(0.0241)	0.392(0.0586)	K562	0.602(0.0912)	0.606(0.0173)	0.535(0.0126)	0.502(0.0363)
GM12878	0.531(0.0161)	0.790(0.0475)	0.495(0.0241)	0.423(0.0479)	GM12878	0.599 (0.0131)	0.746(0.0418)	0.578(0.00937)	0.562(0.0287)
HeLa-S3	0.514(0.0162)	0.531(0.0299)	0.614(0.0751)	0.565(0.0662)	HeLa-S3	0.466(0.0156)	0.440(0.0278)	0.577(0.112)	0.541(0.0431)
IMR90	0.475(0.0148)	0.457(0.0471)	0.488(0.0331)	0.722(0.0592)	IMR90	0.486(0.0187)	0.561(0.0321)	0.561(0.0321)	0.712(0.0674)
